# Spatial distribution of immune cells and their proximity to STING^+^ cells are associated with survival in glioblastoma

**DOI:** 10.1002/ctm2.70187

**Published:** 2025-01-24

**Authors:** Hanwool Jeon, Hayeong Kang, Jihyun Im, Suin Jo, Hyunchul Jung, Moinay Kim, Jae Hyun Kim, Eunyeup Lee, Soyoung Kim, Jeong Hoon Kim, Chang‐Ki Hong, Young‐Hoon Kim, Sang Woo Song, Jinha Park, Sang‐Yeob Kim, Seungjoo Lee

**Affiliations:** ^1^ Department of Neurological Surgery, Asan Medical Center University of Ulsan College of Medicine Seoul Republic of Korea; ^2^ Translational Biomedical Research Group, Asan Institute for Life Sciences Asan Medical Center Seoul Republic of Korea; ^3^ Bio‐Medical Institute of Technology University of Ulsan College of Medicine Seoul Republic of Korea; ^4^ PrismCDX Co., Ltd., Hwaseong‐si Hwaseong Republic of Korea; ^5^ Department of Neurosurgery, Dongsan Medical Center Keimyung University College of Medicine Daegu Republic of Korea; ^6^ Asan Institute for Life Science Asan Medical Center Seoul Republic of Korea

**Keywords:** glioblastoma, multiplex IHC, spatial analysis, tumour immune microenvironment

1

Dear Editor,

Glioblastoma (GBM), the most aggressive malignant tumour, is increasingly treated with immunotherapy.^1^, ^2^, ^3^ The stimulator of interferon genes (STING) pathway^4^ is key to tumour immunity and a studied target for immunotherapy.^5^ This study explores the immune landscape of GBM, focusing on spatial relationships between tumour‐associated immune cells (TAICs)^6^ and STING‐expressing cells, uncovering patterns linked to prognosis.

We studied 14 recurrent GBM patients using protein composition and Gene Ontology (GO) analysis and analyzed immune pathways in 69 newly diagnosed GBM patients undergoing standard therapy. Spatial analysis of cells was performed using QuPath, CytoMAP, and R, as illustrated in Figure 1A. From our proteomic analysis, we identified protein groups with marked upregulation in patients with favourable responses. A total of 99 proteins were highly upregulated in the favourable group, while 170 were more pronounced in the unfavourable group (Figure 1B). Clusters of downregulated and upregulated proteins were identified using the Benjamini‐Hochberg False Discovery Rate. The expression of **TMEM173**, the gene encoding STING, was significantly elevated in the favourable group, as measured by protein expression using a mass spectrometer. The results were represented as an abundance ratio, and statistical significance was confirmed using the adjusted p‐value. (Figure 1C). GO analysis showed that upregulated proteins in the favourable group were mainly linked to immune response activation. (Figure 1D). Pathways such as ‘regulation of innate immune response’ and ‘phagocytic respiratory burst’ were strongly linked to innate immune activation (Figure 1E). Additionally, pathways like ‘cell surface receptor signalling in immune response’, ‘T cell migration’ and ‘positive regulation of T cell receptor signalling’ were significantly linked to immune response activation (Figure 1F). *p*‐Values were transformed to ‐log10 for statistical significance, with values above 1.3 considered significant. Table S1 provides the GO categories associated with immune response activation.

**FIGURE 1:**
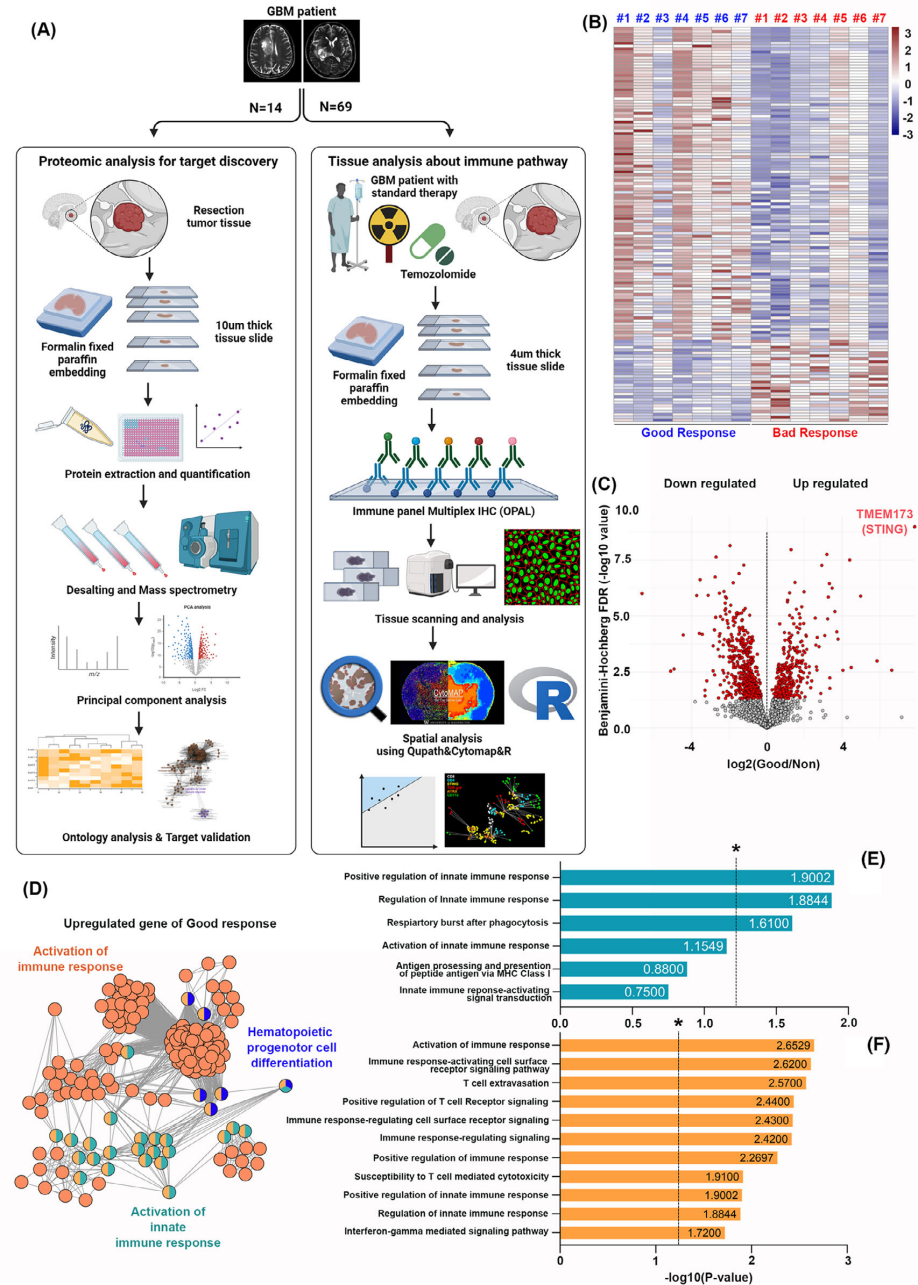
Proteomic analysis of glioblastoma (GBM) related to the immune environment. (A) Overview of the proteomic and immune pathway analyses conducted using multiplex IHC techniques. (B) Heatmap showing the expression levels of 269 proteins in samples from 14 recurrent GBM patients. Among these, 99 proteins were upregulated in the group with a positive response, while 170 proteins were higher in the poor‐response group. (C) Principal component analysis (PCA) analysis results for the positive‐response group, where TMEM173, the gene encoding STING, was notably elevated. Proteins marked in red indicate statistical significance (*p* < .05). (D) Gene ontology results in the positive‐response group, revealing that most immune pathways are linked to immune activation (highlighted in orange). (E, F) Bar charts from gene ontology analysis illustrate pathways associated with the activation of innate immune response (E) and overall immune response activation (F). The illustration for (A) was created using BioRender.com.

We used multiplex immunohistochemistry (IHC) to analyze immune cell distribution and protein markers in the TME. Markers included CD4^+^ (helper T cells), CD8^+^ (cytotoxic T cells), CD11c^+^ (dendritic cells), TCRγ/δ^+^ (γ/δ T cells), ATRX^+^ (tumour cells) and DAPI^+^ (nuclei), with STING as a primary marker of interest.

We explored correlations between immune cell populations identified by multiplex IHC and patient survival (Figure 2A). Tumour specimens were stained with multiplex immunofluorescence, and a pathologist selected regions of interest (ROIs), which were scanned at high magnification and analyzed with markers including ATRX, CD8, STING, DAPI, CD4, CD11c and TCRγ/δ. (Figure 2B). Multiplex IHC enabled analysis of cell interactions in the tumour microenvironment, quantifying marker expression and its impact on prognosis. Correlation analysis between marker expression and overall survival (OS) revealed a significant positive correlation between STING^+^ cell count and OS (*R* = 0.256, *p* = .036) (Figure 2C). No other markers showed a statistically significant correlation with OS. Linear regression analysis showed an increase in OS with higher STING^+^ cell counts (*β* = 0.481, *p* = .036) (Figure 2D). Table S2 summarizes linear regression results for each marker and OS. When adjusted for progression‐free survival (PFS), increased STING^+^ cell counts were significantly linked to improved OS (*β* = 0.302, *p* = .025, *R* ^2^ = 0.692) (Figure 2G). Table S3 details the results of the analysis correlating cell counts with OS, adjusted for PFS. Comparing patients with high and low STING expression revealed that only the patient with low STING expression experienced recurrence 1.5 years after surgery (Figure 2E). Using QuPath, cell coordinates and phenotypes were mapped for spatial analysis in R. Visualization showed that the patient without GBM recurrence had a higher number of STING‐expressing cells (Figure 2F). These results show that multiplex IHC enables visual comparison of TME cell composition differences between patients with favourable and unfavourable outcomes.

**FIGURE 2:**
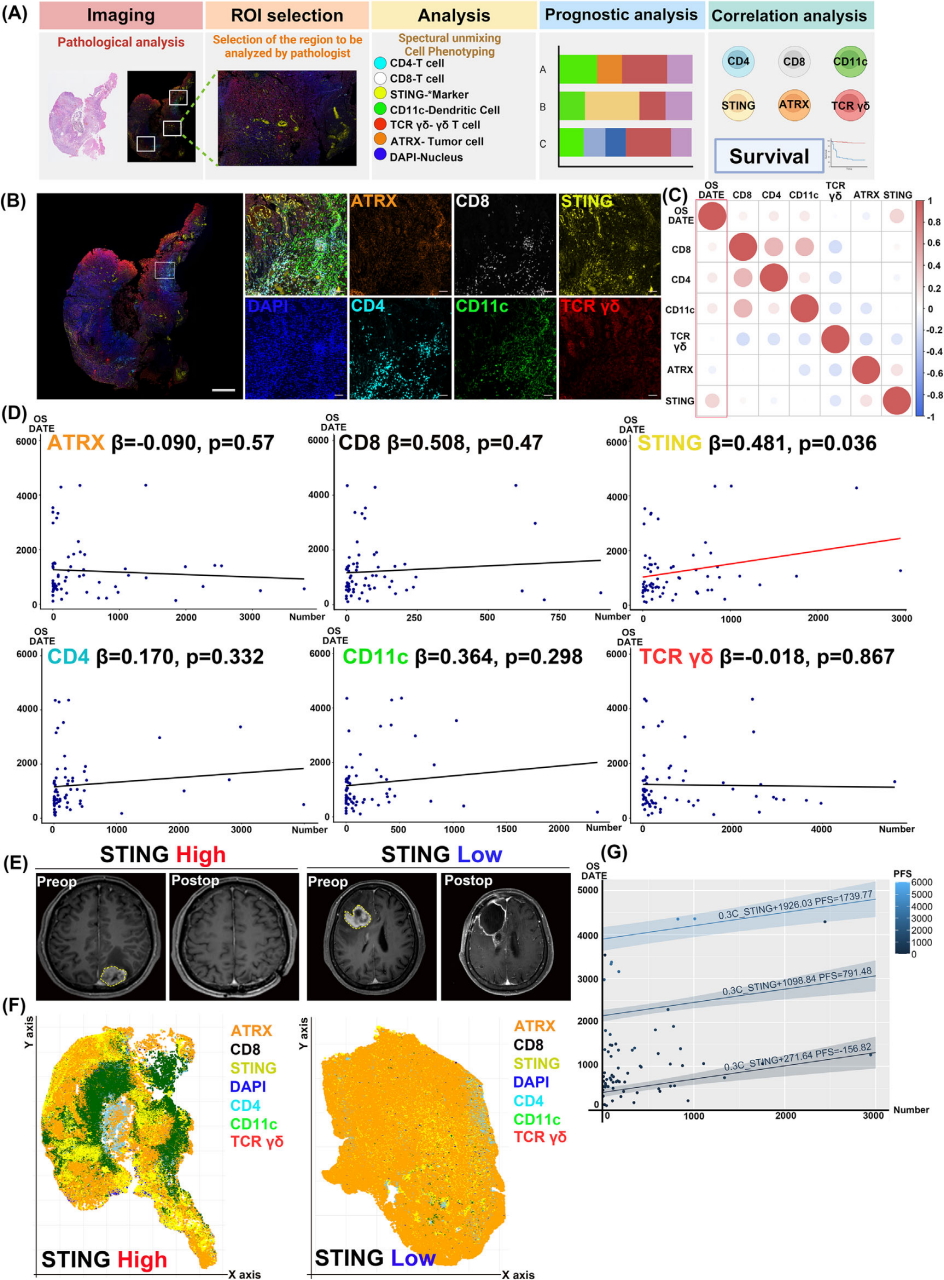
Multiplex immunohistochemistry (IHC) staining of markers and their association with survival. (A) Diagram of the workflow for conducting prognostic analysis through multiplex IHC. (B) Sample images of slides at both low and high magnification, stained with seven distinct markers. (C) Summary heatmap illustrating the relationship between overall survival and the cell counts for each marker. The dot colour represents correlation strength, while the dot size reflects statistical significance. (D) Results from linear regression analysis showing the influence of each marker on overall survival, with STING being the only marker significantly correlated with overall survival (*p* < .05). (E) Brain magnetic resonance imaging (MRI) scans from patients with high versus low STING expression, showing images taken before surgery and 1.5 years afterwards. (F) Positional plots generated in R, based on cell coordinates obtained via QuPath, displaying cells expressing each marker. (G) Two‐dimensional plot of the multiple linear regression analysis depicting the relationship between STING‐expressing cell counts and overall survival (OS). The illustration for (A) was created using BioRender.com.

The STING pathway affects the TME by modulating immunosuppressive and immunoproliferative conditions through T cells and dendritic cells, influencing tumour progression based on the context.^7^ This study analyzed spatial interactions of STING^+^ cells, which are present in hematopoietic, endothelial, epithelial, and fibroblast cells.^8^ STING‐expressing endothelial cells play a key role in promoting immune responses driven by macrophages and help in T cell recruitment across blood vessels.^9^ In the brain's microvascular structure, which comprises the blood‐brain barrier (BBB), type I interferons secreted by endothelial cells enhance immune responses, impacting tumour immunity.^10^ This study specifically targets STING^+^CD4^−^CD8^−^CD11c^−^ATRX^−^TCRγ/δ^−^ cells, which are likely endothelial cells forming the BBB.

We measured mean distances from STING^+^ cells to ATRX^+^ cells, TCRγ/δ^+^ cells, CD4^+^ cells, CD8^+^ cells and CD11c^+^ cells (Figure 3A). The correlation analysis between STING expression and immune markers in our study was conducted using an unsupervised approach. This method allowed us to identify natural patterns and relationships within the data without introducing potential biases from predefined categories or labels. From the patient group, we selected individuals with favourable or poor OS outcomes and generated positional plots of surgical samples (Figure 3B). ROIs chosen by the pathologist were further examined at high magnification, with cell coordinates derived from these plots. Voronoi diagrams were used for selected ROIs to merge clusters and simplify visual representation (Figure 3B). Patients with high STING expression showed CD4^+^ cells near STING^+^ cells and few CD11c^+^ cells, while low STING expression patients had CD11c^+^ cells near STING^+^ cells and scarce CD4^+^ cells (Figure 3B). Notably, the patient with a close spatial relationship between CD4^+^ and STING^+^ cells had a favourable prognosis, with an OS of 3371 days, whereas the patient with CD11c^+^ cells near STING^+^ cells had a poor prognosis, with an OS of 108 days.

**FIGURE 3:**
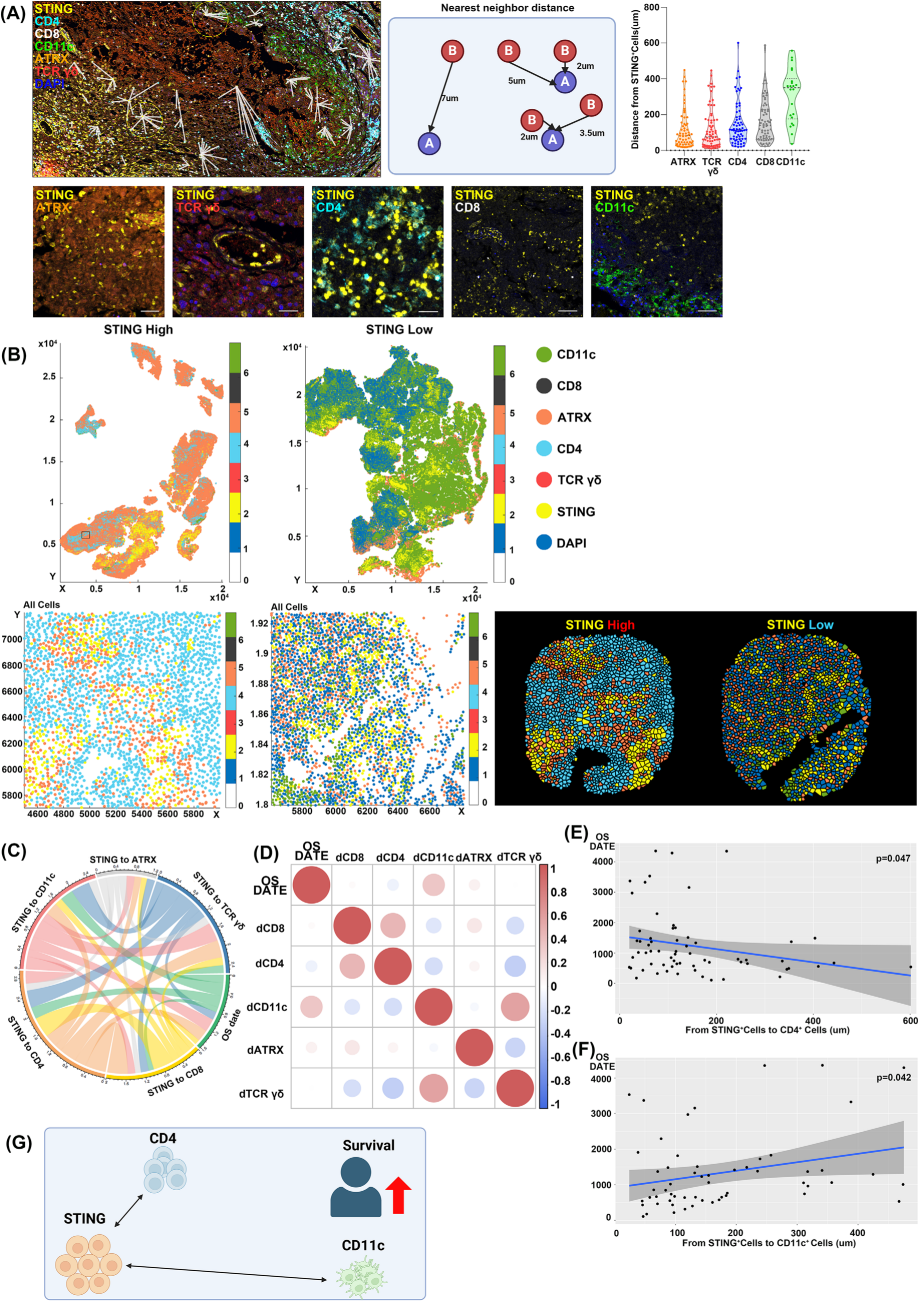
Spatial analysis of STING^+^ cells and nearby immune cells. (A) Diagram showing the method for calculating distances from STING^+^ cells to surrounding target cells. The multiplex image and schematic highlight the measurement of nearest neighbour distances. The violin plot displays the distribution of distances from STING^+^ cells to ATRX^+^ cells, TCRγδ^+^ cells, CD4^+^ cells, CD8^+^ cells and CD11c^+^ cells within the patient group. Although the violin plot is not statistically significant, it provides an overview of the distance distribution between STING^+^ cells and nearby immune cells across patients. Below, multiplex images illustrate the spatial and visual arrangement of cell pairs for each marker. (B) Positional plots for patients with high versus low STING expression visualized with Cytomap, including higher magnification images of regions of interest (ROIs) for each patient. A merged Voronoi map for each ROI is used to reduce visual complexity. (C) Chord diagram depicting the varied relationships between overall survival (OS) and distances from STING^+^ cells to cells expressing each marker, showing that OS is related to distances from STING^+^ cells to CD4^+^ and CD11c^+^ cells. (D) Heatmap summarizing correlations between OS and distances from STING^+^ cells to cells with each marker. For example, “dCD8” refers to the mean distance from STING^+^ cells to CD8^+^ cells. Only statistically significant results (*p* < .05) are displayed. Dot color indicates correlation strength, while dot size reflects statistical significance; red dots indicate positive correlations and blue dots indicate negative correlations. (E) Linear regression analysis showing how distance from STING^+^ cells to CD4^+^ cells affects OS. (F) Linear regression analysis showing how distance from STING^+^ cells to CD11c^+^ cells impacts OS. (G) Diagram illustrating that shorter distances from STING^+^ cells to CD4^+^ cells and longer distances from STING^+^ cells to CD11c^+^ cells are associated with improved survival outcomes. The illustration for (A, G) was created using BioRender.com.

The chord diagram showed a significant link between OS and the mean distances from STING^+^ cells to CD4^+^ and CD11c^+^ cells (Figure 3C). Correlation analysis demonstrated a significant negative correlation between the distance from STING^+^ cells to CD4^+^ cells and OS (R = ‐0.089, *p* = .047). Conversely, a positive correlation between the distance from STING^+^ cells to CD11c^+^ cells and OS was also identified (R = 0.342, *p* = .042) (Figure 3D). Linear regression confirmed that shorter distances between STING^+^ and CD4^+^ cells were linked to lower OS (*p* = .047) (Figure 3E). Likewise, greater distances between STING^+^ and CD11c^+^ cells were associated with increased OS (*p* = .042) (Figure 3F).

These findings indicate a potential association between the spatial relationships of STING^+^ cells and nearby immune cells and survival outcomes in GBM patients. In particular, closer proximity between STING^+^ cells and CD4^+^ cells showed a trend toward improved survival, while greater distance between STING^+^ cells and CD11c^+^ cells appeared to correlate with better outcomes (Figure 3G). Given STING's dual roles in tumour promotion and inhibition, these spatial interactions may contribute to shaping the immune microenvironment in a nuanced manner.

## AUTHOR CONTRIBUTIONS

Hanwool Jeon: Conceptualization, methodology, formal analysis, data curation, funding acquisition and writing and editing; Hayeong Kang: Conceptualization, methodology, formal analysis, data curation and writing and editing; Jihyun Im: Validation, visualization, formal analysis, data curation and writing and editing; Suin Jo: Methodology, validation, visualization, data curation and writing and editing; Hyunchul Jung: Resources; Moinay Kim: Resources; Jae Hyun Kim: Resources; Jeong Hoon Kim: Resources; Chang‐Ki Hong: Resources; Young‐Hoon Kim: Resources; Sang Woo Song: Resources; Jinha Park: Methodology and data curation; Sang‐Yeob Kim: Methodology, data curation, supervision and writing and editing, Seungjoo Lee: Conceptualization, methodology, investigation, validation, formal analysis, data curation, resources, supervision, funding acquisition and writing and editing.

## CONFLICT OF INTEREST STATEMENT

The authors declare no conflict of interest.

## FUNDING INFORMATION

This research was supported by a grant from the Basic Science Research Program, funded by the Korean government's Ministry of Science and ICT (MSIT) (2022R1A2C2011941), Korea Health Technology R&D Project through the Korea Health Industry Development Institute (KHID), funded by the Ministry of Health & Welfare, Republic of Korea (grant number: RS‐2024‐00438911) to Seungjoo Lee. Additionally, support was provided by the Health Fellowship Foundation and the Korean government's Ministry of Science and ICT (2022R1C1C2002698) to Hanwool Jeon.

## Supporting information



Supporting Information
