# Supplementary material for: Spatial distribution of immune cells and their proximity to STING+ cells are associated with survival in glioblastoma
Source: Clin Transl Med. 2025 Jan 24;15(2):e70187. doi: 10.1002/ctm2.70187 (PMC11761387; doi:10.1002/ctm2.70187)
Supplement: Supplementary file 1 — Supporting Information [file CTM2-15-e70187-s001.docx]

***Letter to the Journal CTM2-2024-10-3444 (previous submission number)***

**Supplementary Information**

**Spatial distribution of immune cells and their proximity to STING^+^ cells are associated with survival in glioblastoma**

**Corresponding authors**

^*^**Correspondence to: Seungjoo Lee, MD, PhD**

**Associate professor**

Department of Neurological Surgery, Brain Tumor Center

Asan Medical Center, University of Ulsan College of Medicine

88, Olympic-ro 43-gil, Songpa-gu, Seoul, Republic of Korea

Tel: +82-2-3010-3550; Fax: +82-2-476-6738

E-mail: [changill@gmail.com](mailto:changill@gmail.com); [rghree@amc.seoul.kr](mailto:rghree@amc.seoul.kr)

**This PDF file includes:**

Table

Supplementary methods

Supplementary notes

Supplementary references

**Table 1. List of gene ontology (GO) categories**

| **GOID** | **GO Term** | **-log10 (P-value)** | **Associated Genes Found** |
| --- | --- | --- | --- |
| Activation of innate immune response | | | |
| *GO:0045089* | positive regulation of innate immune response | 1.9002 | [FADD, HCK, NECTIN2, PSMB10, STING1] |
| *GO:0045088* | regulation of innate immune response | 1.8844 | [FADD, HCK, NECTIN2, PSMB10, STING1, TRIM21] |
| *GO:0045728* | respiratory burst after phagocytosis | 1.61 | [HCK] |
| *GO:0002218* | activation of innate immune response | 1.1549 | [HCK, PSMB10, STING1] |
| *GO:0002474* | antigen processing and presentation of peptide antigen via MHC class I | 0.8800 | [PSMB10, SAR1B] |
| *GO:0002758* | innate immune response-activating signal transduction | 0.7500 | [HCK, PSMB10] |
| Activation of immune response | | | |
| *GO:0002253* | activation of immune response | 2.6529 | [HCK, HLA-DPB1, IGHV3-30-5, IGHV5-10-1, IGKV1D-33, NECTIN2, PSMB10, RAB29, STING1, WIPF3] |
| *GO:0002429* | immune response-activating cell surface receptor signaling pathway | 2.6200 | [HCK, HLA-DPB1, IGHV3-30-5, IGHV5-10-1, IGKV1D-33, NECTIN2, PSMB10, RAB29, WIPF3] |
| *GO:0072683* | T cell extravasation | 2.5700 | [CD99, FADD] |
| *GO:0050862* | positive regulation of T cell receptor signaling pathway | 2.4400 | [NECTIN2, RAB29] |
| *GO:0002768* | immune response-regulating cell surface receptor signaling pathway | 2.4300 | [HCK, HLA-DPB1, IGHV3-30-5, IGHV5-10-1, IGKV1D-33, NECTIN2, PSMB10, RAB29, WIPF3] |
| *GO:0002764* | immune response-regulating signaling pathway | 2.4200 | [HCK, HLA-DPB1, IGHV3-30-5, IGHV5-10-1, IGKV1D-33, NECTIN2, PSMB10, RAB29, WIPF3] |
| *GO:0050778* | positive regulation of immune response | 2.2697 | [FADD, HCK, HLA-DPB1, IGHV3-30-5, IGHV5-10-1, IGKV1D-33, NECTIN2, PSMB10, RAB29, STING1, WIPF3] |
| *GO:0060370* | susceptibility to T cell mediated cytotoxicity | 1.9100 | [NECTIN2] |
| *GO:0045089* | positive regulation of innate immune response | 1.9002 | [FADD, HCK, NECTIN2, PSMB10, STING1] |
| *GO:0045088* | regulation of innate immune response | 1.8844 | [FADD, HCK, NECTIN2, PSMB10, STING1, TRIM21] |
| *GO:0060333* | interferon-gamma-mediated signaling pathway | 1.7200 | [HCK, HLA-DPB1, TRIM21] |

**Table 2. Linear regression analysis between cell count and overall survival**

| Value | Data | | β ± SE | *p*-value | R^2^ |
| --- | --- | --- | --- | --- | --- |
|  | X | Y |  |  |  |
| Crude | CD8 | Overall survival | 0.508 ± 0.704 | 0.473 | 0.008 |
|  | CD4 |  | 0.170 ± 0.174 | 0.332 | 0.015 |
|  | CD11c |  | 0.364 ± 0.347 | 0.298 | 0.017 |
|  | TCRgd |  | -0.018 ± 0.108 | 0.867 | 0.000 |
|  | ATRX |  | -0.090 ± 0.157 | 0.567 | 0.005 |
|  | STING |  | 0.481 ± 0.225 | 0.036 | 0.066 |

**Table 3. Linear regression analysis between cell count and overall survival (adjusted for progression-free survival)**

| Value | Data | | β ± SE | *p*-value | R^2^ |
| --- | --- | --- | --- | --- | --- |
|  | X | Y |  |  |  |
| Adjusted for progression-free survival | CD8 | Overall survival | -0.023 ± 0.414 | 0.956 | 0.666 |
|  | CD4 |  | -0.099 ± 0.104 | 0.347 | 0.671 |
|  | CD11c |  | 0.359 ± 0.199 | 0.076 | 0.682 |
|  | TCRgd |  | -0.017 ± 0.063 | 0.783 | 0.666 |
|  | ATRX |  | -0.13 ± 0.09 | 0.154 | 0.676 |
|  | STING |  | 0.302 ± 0.131 | 0.025 | 0.692 |

**Table 4. List of multiplex IHC antibodies**

| **Target** | **Catalog number** | **Concentration** | **company** |
| --- | --- | --- | --- |
| CD11c | Ab52632 | 1:500 | Abcam |
| STING | 19851-1-AP | 1:2000 | Proteintech |
| CD4 | Ab133616 | 1:100 | Abcam |
| TCR g/d | 331202 | 1:100 | Biolegend |
| CD8 | NB100-35729 | 1:100 | NOVUS |
| ATRX | HPA001906 | 1:500 | Sigma |

**SUPPLEMENTARY METHODS**

**Study design**

The study was approved by the Institutional Review Board of Asan Medical Center (IRB 2019-0082, 2017-0665, 2016-1245) and involved patients with recurrent grade IV glioblastoma (GBM) undergoing standard therapy. Eligibility criteria included: (1) age between 19 and 80 years, (2) follow-up MRI scans available, specifically pre- and post-contrast T1-weighted imaging, (3) a confirmed pathological diagnosis of GBM, and (4) concurrent treatment with temozolomide-based chemoradiotherapy. Patients were excluded if MRI scans revealed indistinct recurrent lesions or post-radiotherapy necrosis, if they had a Karnofsky Performance Scale score below 40, or if only minimal tissue samples were available.

**Concurrent Chemoradiation Therapy (CCRT)**
n accordance with CCRT guidelines^1^ at our institution, patients received focal radiotherapy five days a week at a dose of 2 Gy per session for six weeks, reaching a total dose of 60 Gy. Concurrently, temozolomide was given daily at 75 mg/m² during the radiotherapy period. Following a four-week rest, patients began up to six cycles of adjuvant temozolomide, administered over five days every four weeks. The initial dose of temozolomide was set at 150 mg/m², with an increase to 200 mg/m² from the second cycle onward, assuming no adverse effects were observed. Recurrence was defined as a newly appearing or enlarging contrast-enhanced mass larger than 1 x 1 cm, with an increase of more than 25% on MRI scans taken at least 12 weeks after completing CCRT. Strict protocol criteria were followed to exclude cases of pseudo-progression^2^.

**Proteomic analysis cohort**

For proteomic studies, we selected seven patients with a favorable prognosis and seven with poor outcomes, characterized by rapid recurrence and limited survival. Initially, 163 recurrent GBM patients treated with temozolomide from 2010 to 2016 were considered. Seventy-one patients were excluded due to stereotactic biopsy, lack or follow-up or partial resection. Of the remaining patients, an additional 20 patients did not meet quality control criteria for specimen analysis, leaving a final cohort of 72 patients, ranked by survival duration. The seven patients with the longest survival and the seven with the shortest survival were selected. Baseline clinical features were comparable between groups, with survival duration as the main variable.

**Response assessment**

MRI scans were performed every two or three months to monitor patients. Tumor progression was evaluated either through additional surgery or combined clinical and radiological assessments, determined by consensus among three neuro-oncologists and one radiologist, each with over a decade of experience. Evaluations followed the (RANO) Response Assessment in Neuro-Oncology criteria^3^. Progression was classified as (1) enhancing local progression within 3 cm of the primary site, (2) diffuse, non-enhancing progression with stable local contrast but FLAIR hyperintensity extending beyond 3 cm, or (3) distant progression showing FLAIR hyperintensity over 3 cm from the primary site. Determination of progression patterns required consensus from two neuroradiologists. Progression-free survival (PFS) was calculated from the start of secondary temozolomide therapy until radiologic evidence of disease progression or death. Overall survival (OS) was measured from the initiation of secondary temozolomide treatment until death.

**Proteomic Analysis of GBM Tissue via Mass Spectrometry**

For protein detection, paraffin-embedded tissue samples were sectioned into 10 μm-thick slides. The sections were collected in a microcentrifuge tube, combined with heptane, and allowed to incubate at room temperature for an hour. Then, methanol was added, followed by 10 seconds of vortexing and centrifugation. After carefully removing the supernatant, the pellet was air-dried for 5 minutes and then vortexed with EXB Plus extraction buffer and beta-mercaptoethanol. Following a 5-minute incubation, the sample was vortexed again and heated at 100°C for 20 minutes. It was then incubated at 80°C for two hours at 750 rpm, briefly cooled at 4°C, and centrifuged at 14,000 × g for 15 minutes at 4°C. The supernatant was transferred to a new tube, and the protein concentration was determined using the BCA assay. Once the protein pellet was dissolved, reduction, alkylation, and tryptic digestion were conducted, followed by peptide analysis using LC-HRMS.

**Multiplex IHC analysis cohort**

The 103 patients who was treated with CCRT

from 2015-2020 at the subject hospital

36 patients excluded (partial resection, closed needle biopsy, follow-up loss, Tissue loss)

69 patients treated with temozolomide

69 patients included

(Multiplex IHC analysis)

For the multiplex IHC analysis, 103 patients treated with temozolomide for recurrent GBM from 2015 to 2020 were identified. Thirty-six patients were excluded due to partial resection, stereotactic biopsy, loss to follow-up, or tissue loss, and immune cell distribution was analyzed in the remaining 69 patients.

**Multiplex immunofluorescence staining**

Tissue sections, 4 μm thick, were cut from paraffin-embedded FFPE blocks. The slides were first heated in a 60°C dry oven for at least an hour before proceeding to staining using the Leica Bond Rx™ automated stainer (Leica Biosystems) for multiplex immunofluorescence. After a 30-minute incubation, the slides were deparaffinized with Leica Bond Dewax Solution (#AR9222, Leica Biosystems). Antigen unmasking was performed with Bond Epitope Retrieval 2 (#AR9640, Leica Biosystems) for 30 minutes. The primary antibody CD11c (ab52632, Abcam, 1:500 dilution) was applied first for 30 minutes, followed by detection with Polymer HRP Ms^+^Rb (ARH1001EA, AKOYA Biosciences) for 10 minutes. CD11c was visualized with Opal 520 TSA (1:150 dilution) for 10 minutes. To prepare for the next staining, Bond Epitope Retrieval 1 (#AR9961, Leica Biosystems) was applied for 20 minutes to remove bound antibodies.

Subsequent staining included STING (19851-1-AP, Proteintech, 1:2000), which was detected with Polymer HRP Ms^+^Rb and visualized with Opal 570 TSA (1:150 dilution) for 10 minutes. CD4 (ab133616, Abcam, 1:100 dilution) followed, with the same detection method and visualization with Opal 480 TSA (1:150 dilution). After CD4, TCR γ/δ (331202, BioLegend, 1:100 dilution) was applied and visualized with Opal 690 TSA (1:150 dilution). The final antibodies included CD8 (NB100-35729, Novus Biologicals, 1:100 dilution) visualized with Opal 780 TSA, and ATRX (HPA001906, Sigma, 1:500 dilution) visualized with Opal 620 TSA. Each antibody application was followed by Bond Epitope Retrieval 1 to clear previously bound antibodies. Nuclear staining was completed with DAPI, and the slides were mounted with ProLong Gold antifade reagent (P36934, Invitrogen).

**Image acquisition and spatial analysis**

Slides were scanned with the Vectra Polaris Automated Quantitative Pathology Imaging System (Akoya Biosciences, Marlborough, MA), and images were processed using Inform 2.4 software (Akoya Biosciences, Marlborough, MA) and TIBCO Spotfire™ (TIBCO, Palo Alto, CA). For accurate separation of fluorescent signals, we used reference slides representing each emission spectrum alongside unstained tissue slides. Each uniquely stained section contributed to building a comprehensive spectral library of fluorophores essential for multispectral analysis. This library served as the benchmark for quantifying targets, allowing the intensity of each fluorescence signal to be extracted through linear unmixing. Cells were identified by recognizing nuclear markers (DAPI staining). All immune cell groups in each panel were quantified and classified using the segmentation and phenotyping tools within Inform software. Batch analysis on selected regions of glioblastoma tissue was conducted using a consistent algorithm derived from representative images. After merging the data in RStudio version 1.3.1073, cell counts by phenotype across patient groups were assessed in TIBCO Spotfire™.

To perform spatial analysis on tumor-infiltrating immune cells and STING^+^ biomarker cells, we mapped the coordinates of each cell within the tissue using QuPath software (<https://qupath.github.io/>). Based on staining patterns, we segmented the cells and carried out spatial analysis with these coordinates. CytoMAP12, developed in MATLAB version 2018b (MathWorks), was used for spatial mapping; detailed guidance on CytoMAP’s functions is available in the online user manual. Coordinates from QuPath were imported into CytoMAP via a .csv file, and tools like cell clustering, statistical summaries, and annotation features were used to evaluate spatial data. Following these steps, annotated cell coordinates were used to generate a Voronoi plot through Python.

**Statistical analysis**

All statistical analyses were conducted using R software (version 4.3.1; R Foundation for Statistical Computing). Data visualization was achieved with the 'ggplot' and 'ggcorrplot' packages within RStudio. All variables in the analysis were treated as continuous. The associations among cell counts, distances between cells, and overall survival (OS) were evaluated using Pearson correlation and linear regression methods. In the generated correlogram, the size of each circle reflects the strength of the correlation coefficient, while the color represents the direction of the correlation. The linear regression model was applied to determine the slope and intercept. To analyze the relationships among cell counts, cell-to-cell distances, and OS, multiple linear regression was utilized, adjusting for progression-free survival (PFS) as a continuous variable. The blue line in the graphs represents the line of best fit from the linear regression, and the shaded gray area around this line shows the standard error. Statistical significance was set at p < 0.05 for all results.

**SUPPLEMENTARY NOTES**

Glioblastoma (GBM) develops an immunosuppressive tumor microenvironment (TME), which includes a mixture of immunosuppressive cytokines produced by tumor cells along with various microglia and tumor-associated immune cells (TAICs)^4^. Immunosuppressive cells within this TME interact with the tumor through specific receptors, aiding in tumor growth and immune evasion.^5^. The main lymphoid cells in the GBM TME are T cells, including cytotoxic T cells (CD8^+^), helper T cells (CD4^+^), and regulatory T cells (Tregs)^6^. CD8^+^ T cells destroy abnormal cells, including cancer cells, while CD4^+^ T cells support the function of CD8^+^ cells. Tregs, however, inhibit immune responses, creating an immunosuppressive environment by limiting CD8^+^ T cells and antigen-presenting cells (APCs).^6, 7^.

STING is found in hematopoietic cells and other cell types, including endothelial, epithelial, and fibroblast cells^8^. STING-expressing endothelial cells play a key role in promoting immune responses driven by macrophages and help in T cell recruitment across blood vessels^9^. In the brain’s microvascular structure, which comprises the blood-brain barrier (BBB), type I interferons (IFNs) secreted by endothelial cells enhance immune responses, impacting tumor immunity^10^. This study specifically targets STING^+^CD4-CD8-CD11c-ATRX-TCRγ/δ- cells, which are likely endothelial cells forming the BBB.

The STING pathway is essential for the production of type I IFNs. When DNA is detected through STING, dendritic cells (DCs) release type I IFNs, promoting cross-priming of DCs and T cells^11^. In CD4^+^ T cells, the STING pathway enhances anti-tumor immunity through type 1 helper and IL-9-producing T cells^12^. This aligns with our findings that close spatial proximity between STING^+^ cells and CD4^+^ T cells is linked to a better prognosis in GBM patients. Conversely, STING also activates IDO, which enhances Treg activity and contributes to a tolerogenic TME^13^, indicating that STING’s role in promoting or inhibiting tumor growth depends on its interactions within the TME.

Dendritic cells (DCs) are diverse APCs with different subtypes, each with distinct transcriptional programs and functions. TIDCs play a critical role in immune surveillance within cancers, and their behavior in the TME is influenced by tumor growth, blood supply, and hypoxia^11^. nterestingly, we observed that STING^+^ cells close to CD11c^+^ cells, typically DCs, were associated with a poor prognosis, which contrasts with the known role of STING in supporting anti-tumor immunity within DCs^14^. Generally, a higher frequency of TIDCs suggests a favorable prognosis; however, TIDCs may have dual effects, either promoting or suppressing anti-tumor immunity, based on their environment ^15^. Research has shown that the STING pathway in myeloid dendritic cells triggers IDO, resulting in a strong tolerogenic response^16^. TIDCs may be linked to either positive or negative prognoses, depending on their maturation stage. This variability has been noted in cancers like colorectal, lung, and ovarian cancer but has not been observed in GBM^15^. The dual impact of TIDCs in tumor immunity is likely due to soluble factors released by tumors, which can hinder TIDC maturation and differentiation^17, 18^. Immune cells within the TME function differently based on environmental factors and their chemical and physical interactions with nearby cells, affecting disease progression. Future studies should consider not only each cell’s biochemical function in anti-tumor immunity but also their spatial relationships and physical interactions.

The STING pathway has long been recognized in cancer biology, and there is ongoing interest in targeting STING for immunotherapy. Studies evaluating STING agonists in mouse models of melanoma, colon cancer, and breast cancer have shown promising therapeutic effects^14^. However, these agonists have not been as successful in human trials^19^. Nonetheless, since type I IFN is linked to increased tumor T cell infiltration and improved survival, STING activation in the TME holds potential as a prognostic marker^14, 20^. Research should continue to explore STING as a cancer therapy target, focusing on balancing STING activity in the TME to effectively induce type I IFN and enhance immune cell activation for anti-tumor responses. Stratifying GBM patients for immunotherapy based on STING status and immune cell interactions may improve treatment outcomes^21^.

Immunotherapy, which activates the immune system to target cancer, has proven effective in several cancers, including melanoma and non-small cell lung cancer. GBM, however, presents unique challenges due to high intra-tumoral and inter-tumoral heterogeneity. This cellular diversity allows GBM to evade immune responses, metastasize, and resist treatments^22^. Clinical trials have tested immunotherapy for GBM, including studies on the EGFRvIII-targeted vaccine rindopepimut for newly diagnosed GBM and the checkpoint inhibitor nivolumab for recurrent cases; however, neither trial produced favorable results^23^.

IFN-γ is another potential target for GBM immunotherapy due to its anti-proliferative, anti-angiogenic, and pro-apoptotic effects, which enhance anti-tumor immunity by increasing antigen presentation^24^. However, in GBM, low levels of IFN-γ may upregulate PD-L1 and increase Tregs, allowing immune evasion^25^. This highlights why previous GBM immunotherapy attempts have been limited. A personalized approach involving marker expression analysis and the assessment of each patient's tumor tissue composition is necessary to address GBM’s unique resistance to treatment and to better understand tumor biology.

Tumor tissue is spatially organized, with the spatial arrangement of cells playing a key role in disease behavior and survival predictions. Analyzing the spatial distribution of malignant cells and TAICs can reveal interactions and provide insights for prognostic predictions based on these patterns. Spatial organization studies have been done in cancers like NSCLC, breast cancer, and liver cancer. In GBM, spatial analysis has also shown that a strong correlation between CD73 in tumor cells and CD39 in microglia is linked to poorer outcomes^26^. However, GBM’s spatial organization is not fully understood. This study analyzes cell populations in the GBM TME and examines survival by assessing distances between specific cells, such as STING^+^ cells and other TAICs.

Spatial analysis with multiplex immunohistochemistry (IHC) images visually captures cell interactions, quantifies immune subsets, and reveals immune-tumor dynamics^27^. This method helps to identify the composition and location of tumor-infiltrating and surrounding immune cells, which may be predictive of treatment responses. Spatial analysis can be categorized as either examining statistical correlations of cell types in images or measuring physical distances between cells^22^. This study contributes by analyzing statistical and spatial relationships among various cell types in the GBM TME.

The shift in cancer therapy is increasingly towards personalized treatment, focusing on therapies targeting specific tumor markers. Ongoing research on GBM immunotherapy has yet to produce reliable biomarkers for predicting therapy success or patient outcomes. Challenges stem from the complex immune characteristics of the brain and the heterogeneous nature of GBM. Developing effective treatments and predicting outcomes in GBM requires a detailed understanding of interactions between tumor tissue and nearby immune cells, paving the way for tailored treatment strategies.

**SUPPLEMENTARY REFERENCES**

1. Stupp R*, et al.* Radiotherapy plus concomitant and adjuvant temozolomide for glioblastoma. *The New England journal of medicine* **352**, 987-996 (2005).

2. Wen PY*, et al.* Updated response assessment criteria for high-grade gliomas: response assessment in neuro-oncology working group. *J Clin Oncol* **28**, 1963-1972 (2010).

3. Chinot OL, Macdonald DR, Abrey LE, Zahlmann G, Kerloëguen Y, Cloughesy TF. Response Assessment Criteria for Glioblastoma: Practical Adaptation and Implementation in Clinical Trials of Antiangiogenic Therapy. *Current Neurology and Neuroscience Reports* **13**, 347 (2013).

4. Brown NF, Carter TJ, Ottaviani D, Mulholland P. Harnessing the immune system in glioblastoma. *Br J Cancer* **119**, 1171-1181 (2018).

5. Fanelli GN*, et al.* Decipher the Glioblastoma Microenvironment: The First Milestone for New Groundbreaking Therapeutic Strategies. *Genes (Basel)* **12**, (2021).

6. Andersen RS, Anand A, Harwood DSL, Kristensen BW. Tumor-Associated Microglia and Macrophages in the Glioblastoma Microenvironment and Their Implications for Therapy. *Cancers (Basel)* **13**, (2021).

7. Strepkos D, Markouli M, Klonou A, Piperi C, Papavassiliou AG. Insights in the immunobiology of glioblastoma. *J Mol Med (Berl)* **98**, 1-10 (2020).

8. Digre A, Lindskog C. The Human Protein Atlas-Spatial localization of the human proteome in health and disease. *Protein Sci* **30**, 218-233 (2021).

9. Anastasiou M*, et al.* Endothelial STING controls T cell transmigration in an IFNI-dependent manner. *JCI Insight* **6**, (2021).

10. Duarte N, Shafi AM, Penha-Gonçalves C, Pais TF. Endothelial type I interferon response and brain diseases: identifying STING as a therapeutic target. *Front Cell Dev Biol* **11**, 1249235 (2023).

11. Vatner RE, Janssen EM. STING, DCs and the link between innate and adaptive tumor immunity. *Mol Immunol* **110**, 13-23 (2019).

12. Benoit-Lizon I*, et al.* CD4 T cell-intrinsic STING signaling controls the differentiation and effector functions of T(H)1 and T(H)9 cells. *J Immunother Cancer* **10**, (2022).

13. Lemos H*, et al.* STING Promotes the Growth of Tumors Characterized by Low Antigenicity via IDO Activation. *Cancer Res* **76**, 2076-2081 (2016).

14. Corrales L, Gajewski TF. Molecular Pathways: Targeting the Stimulator of Interferon Genes (STING) in the Immunotherapy of Cancer. *Clin Cancer Res* **21**, 4774-4779 (2015).

15. Karthaus N, Torensma R, Tel J. Deciphering the message broadcast by tumor-infiltrating dendritic cells. *Am J Pathol* **181**, 733-742 (2012).

16. Huang L*, et al.* Cutting edge: DNA sensing via the STING adaptor in myeloid dendritic cells induces potent tolerogenic responses. *J Immunol* **191**, 3509-3513 (2013).

17. Gottfried E, Kreutz M, Mackensen A. Tumor-induced modulation of dendritic cell function. *Cytokine Growth Factor Rev* **19**, 65-77 (2008).

18. Menetrier-Caux C*, et al.* Inhibition of the differentiation of dendritic cells from CD34(+) progenitors by tumor cells: role of interleukin-6 and macrophage colony-stimulating factor. *Blood* **92**, 4778-4791 (1998).

19. Corrales L*, et al.* Direct Activation of STING in the Tumor Microenvironment Leads to Potent and Systemic Tumor Regression and Immunity. *Cell Rep* **11**, 1018-1030 (2015).

20. Jeon H*, et al.* Proteomic analysis predicts anti-angiogenic resistance in recurred glioblastoma. *J Transl Med* **21**, 69 (2023).

21. Lanng KRB, Lauridsen EL, Jakobsen MR. The balance of STING signaling orchestrates immunity in cancer. *Nat Immunol* **25**, 1144-1157 (2024).

22. de Souza N, Zhao S, Bodenmiller B. Multiplex protein imaging in tumour biology. *Nat Rev Cancer* **24**, 171-191 (2024).

23. Weller M*, et al.* Rindopepimut with temozolomide for patients with newly diagnosed, EGFRvIII-expressing glioblastoma (ACT IV): a randomised, double-blind, international phase 3 trial. *Lancet Oncol* **18**, 1373-1385 (2017).

24. Zaidi MR, Merlino G. The two faces of interferon-γ in cancer. *Clin Cancer Res* **17**, 6118-6124 (2011).

25. Zhu VF, Yang J, Lebrun DG, Li M. Understanding the role of cytokines in Glioblastoma Multiforme pathogenesis. *Cancer letters* **316**, 139-150 (2012).

26. Coy S*, et al.* Single cell spatial analysis reveals the topology of immunomodulatory purinergic signaling in glioblastoma. *Nat Commun* **13**, 4814 (2022).

27. Kumar G, Pandurengan RK, Parra ER, Kannan K, Haymaker C. Spatial modelling of the tumor microenvironment from multiplex immunofluorescence images: methods and applications. *Front Immunol* **14**, 1288802 (2023).
